# Evaluation of an optimal receiver operating characteristic procedure

**DOI:** 10.1186/1753-6561-3-s7-s56

**Published:** 2009-12-15

**Authors:** Neal Jeffries, Gang Zheng

**Affiliations:** 1 Office of Biostatistics Research, Division of Population and Prevention Studies, National Heart, Lung, and Blood Institute, Bethesda, Maryland 20892, USA

## Abstract

Lu and Elston have recently proposed a procedure for developing optimal receiver operating characteristic curves that maximize the area under a receiver operating characteristic curve in the setting of a predictive genetic test. The method requires only summary data, not individual level genetic data. In an era of increased data sharing, we investigate the performance of this algorithm when individual level genetic data are available and compare this approach to more standard receiver operating characteristic curve-building methods.

**Conclusion:**

Though the Lu-Elston method can produce an optimal area under the curve under some assumptions, the method typically has little advantage over standard multivariable logistic methods when data are available. Also, the standard approach easily allows comparison of nested models via likelihood ratio tests and incorporation of covariates - the Lu-Elston approach is shown to have some difficulties with such analyses. These conclusions are based on evaluations using the Genetic Analysis Workshop 16 rheumatoid arthritis data set.

## Background

Lu and Elston [1] present an approach to constructing optimal area under the curve (AUC) curves, applicable to case-control studies that does not require the availability of a data set. The method may be based solely upon summary information of marker-specific allele frequencies in cases and controls, penetrance, and disease prevalence. In this approach one can construct multivariable predictive models of disease without knowing the joint distribution of the markers, using an assumption of no interactions between markers. Further, the method is optimal in the sense that the area under the receiver operator characteristic (ROC) curve is maximized. The authors provide a more complex extended model involving linkage disequilibrium (LD) correlations and haplotype frequency estimation that allows for interactions among markers; however, evaluations using this approach are not pursued in this brief report because we would not expect our conclusions to change qualitatively.

Here we assess how this method performs when a case-control data set is available and therefore many methods are available for constructing ROC curves based on the joint distribution of markers. In such situations we examine the extent to which the approach remains optimal and how it may be extended with respect to marker selection and incorporation of covariates. These issues are examined using a small subset of markers drawn from the Genetic Analysis Workshop 16 (GAW16) rheumatoid arthritis (RA) data.

## Methods

The Lu-Elston approach is based on disease-specific genotype frequencies, , where *D *denotes a case, *i *denotes a single-nucleotide polymorphism (SNP), and *j*_*i *_denotes a genotype for that SNP, e.g., Aa. Lu-Elston show how these can be obtained from existing publications with information about population genotype frequencies and genotype relative risks or odds ratios. It is important to note that this can be obtained without individual data. Then one can produce multilocus genotype probabilities from the individual SNP probabilities by assuming independence between markers:

where a small number of loci (e.g., *n *= 6) are considered. In this case *k *is between 1 and *K*, where *K *is the total number of possible genotypes. In the example of six SNPs with unspecified inheritance mode, this could correspond to 3^6 ^= 729 possible genotypes. The multiplicative independence assumption allows one to assign this probability though no one in any data set might ever show this particular genomic combination. With  denoting a control participant, one forms the likelihood ratios

The optimal ROC curve is then obtained by sorting the *LR*_*k *_from largest, *LR*_(*K*) _to smallest *LR*_(1)_. By varying cutoffs along the range of *LR*_(*k*)_, one can obtain true positive rates (TPRs) and false positive rate (FPRs) for computing the AUC. For a fixed threshold c,

and the AUC is computed using the trapezoidal rule as in Lu and Elston [1].

## Results

### Application to GAW16

The Lu-Elston approach uses a set of markers to categorize individuals as testing positive or testing negative. The approach is not designed for biomarker selection/discovery, but to construct a ROC curve with a given set of predetermined markers. For this data set the predetermined SNP markers studied were rs6457617, rs2476601, rs7574865, rs1061622, rs2073838, rs1248696, corresponding to *MHC, PTPN22, STAT4, TNFRSF1B, SLC22A4*, and *DLG5 *genes, respectively. The first three were chosen because they were linked to RA [2]. The last three SNPs were chosen because 1) they were among the SNPs selected for an RA candidate gene study [3] and 2) among the candidate SNPs only these three appear to be included in this Illumina data set. Thus, all six SNPs were chosen independently from the GAW16 data under consideration. Applying the Lu-Elston approach by taking the genotype frequencies,  from the RA data set yields an ROC curve with AUC = 0.7504. Without a data set, one could have used estimates of prevalence, allele frequencies, and published log-odds ratios to derive genotype frequencies for cases and controls as described by Lu-Elston. However, because the purpose of using these estimates is to obtain genotype frequencies for cases and controls, it is easier to estimate  directly from the data at hand. Further, using the data at hand promotes comparability between this approach and the logistic regression approaches.

The AUC is a global summary measure of how the FPRs and TPRs change as the cutoff for declaring a positive test is varied. It will be compared to the AUC obtained using conventional logistic regression methods and the same set of predetermined SNPs. Starting with a ROC curve either the Lu-Elston or logistic regression method can be used to develop a diagnostic test.

### Logistic regression as an alternative to Lu-Elston

Given a case-control data set, the Lu-Elston ROC and underlying TPRs and FPRs may be easily obtained through conventional methods because

and  may be obtained from a univariate logistic regression of case/control status on marker *i *(i.e., treating the marker as a factor with three levels corresponding to the AA, Aa, and aa genotypes) or from simple observation of marker-specific disease rates. In all equations the probabilities correspond to empirically observed probabilities from the data set at hand. Then from Eqs. (1), (2), and (4) we see the empirically observed likelihood ratio, *LR*_(k)_, is a function of fitted probabilities from six univariate logistic regression models and a ratio of controls to cases in the data set,

Given that the Lu-Elston ROC may be exactly reproduced from a series of univariate regression analyses, this raises the question of whether a single multivariate regression model may produce better discrimination. It might be expected that the fitted probabilities from six univariate models are a special case of the fitted probabilities available from a multivariate model so a higher AUC could be obtained in a less restricted multivariate model. Alternatively, Lu and Elston argue their model is optimal in that it should have the highest AUC value. It is in fact the case that no broad generalizations can be made--in some cases the optimal Lu-Elston method as described above will produce an AUC exceeding that constructed through a simple multiple logistic regression of the same factors, and in other situations the Lu-Elston method will perform worse. This arises because different assumptions may be made regarding *P*(*G*_*k*_|*D*) and the collection of genotypes under consideration may differ.

**Figure 1:**
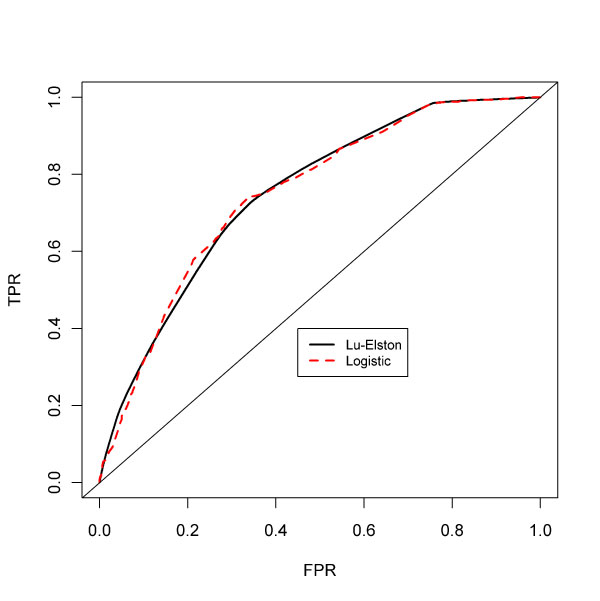
**Lu-Elston and multivariate logistic ROCs for six SNPs**. Lu-Elston ROC = 0.7504, logistic ROC = 0.7503.

As an example, one may perform multivariate logistic regression of case/control status on 6 explanatory factors--each factor corresponds to one of the markers and each factor has three levels. With an assumption of no interactions between markers, this corresponds to a model with an intercept term and 12 other coefficients. The corresponding ROC curve (composed by ordering the predicted *P*(*D*|*G*_*k*_) values from the model) is shown in Figure 1 along with the original Lu-Elston ROC curve.

The two curves are quite close though the logistic curve has a slightly lower computed AUC--0.7503 compared with 0.7504. This slight decrease associated with the logistic may not be generalized. For example, if only five markers are used (excluding the third marker, rs7574865) then the Lu-Elston AUC is 0.7487; the multivariate model AUC is 0.7490. Of course, these AUCs are identical for practical purposes; the comparisons show neither approach has uniformly higher AUCs.

For the case of six markers, the Lu-Elston curve is based on 3^6^, or 729 possible genotypes while the logistic curve is based on the unique 178 genotypes that are observed as determined by the six SNPs. However, the approaches produce similar results and this might be expected if the relation in Eq. (1) is approximately true.

As an aside we note that given a data set with individual-specific genotypes, the AUC can be maximized beyond what has been shown thus far by applying the Lu-Elston idea to the distinct genotypes observed (in this case 178), rather than those derived using the independence assumption in Eq. (1). One may construct the ROC curve using likelihood ratios derived not from the independence assumption but by the observed values of

where *P*(*D*|*G*_*k*_) is the empirically observed proportion of cases among those with genotype *G*_*k *_Using Eq. (6), one can derive an empirical ROC of 0.793. However, such an approach likely yields an overfitted model. As an example, a genotype with two cases and no controls would have an infinitely large *LR*_(*K*) _with estimated sensitivity of 100%--a figure that is not likely to be reproduced in a follow-up study with more individuals having that genotype.

### Model fitting aspects

The Lu-Elston paper discusses how to choose among optimal ROC models based on different collections of SNPs from a common data set. They propose calculation of

where *Var*(*A*_2 _- *A*_1_) = *Var*(*A*_2_)+ *Var*(*A*_1_) - 2*Cov*(*A*_2_, *A*_1_), *A*_2 _and *A*_1 _represent the different optimal AUCs corresponding to the two collections of SNPs, and the variance term in the denominator would be estimated by a bootstrap approach. They propose comparing the resulting *Z*-statistic to a standard normal distribution to assess whether one collection represents a significant improvement over the other. However, in the context of nested collections when one collection properly contains all the SNPs in the other collection, such a comparison likely produces *p*-values that are incorrect. This follows because if the second collection properly contains the first, then the optimality theorem of Lu-Elston dictates that *A*_2 _(the AUC associated with the second collection) must exceed *A*_1 _and *Z *in Eq. (7) is necessarily positive. Therefore, the evaluation of *Z *by a standard normal distribution is not appropriate.

As a conventional alternative, a likelihood-ratio test may be used with the multivariate logistic regression approach to determine whether an additional marker would improve the model.

To evaluate which of the methods (bootstrap or multivariable logistic likelihood-ratio test) had appropriate type I error behavior when adding an unrelated SNP, we sampled 1445 markers drawn from those chromosomes that hold none of the original six markers and were spaced at roughly equidistant intervals for a given chromosome. Originally, 2000 such markers were drawn but only 1445 met quality control and minor allele frequency conditions to ensure that bootstrap samples would generate all three genotypes. Our assumption is that few, if any, of these markers are strongly related to arthritis.

As expected, the bootstrap approach did not perform well because a standard normal distribution centered about 0 is ill-suited for evaluating a test statistic that is necessarily non-negative (i.e., *A*_2 _≥ *A*_1_). The Kolmogorov-Smirnov *p*-value for testing if the 1445 test statistics followed a standard normal distribution was *p *< 10^-15^. On the other hand, the likelihood ratio test performed appropriately for the multivariate logistic regression approach. The *p*-value for testing whether the 1445 test statistics followed a χ^2 ^distribution with two degrees of freedom was *p *= 0.55. The likelihood ratio test incorporates a genomic control correction [4] for population stratification that is achieved by dividing all the 1445 log-likelihood ratio test statistics by the ratio of the median test statistic value and the median value of a χ^2 ^distribution with two degrees of freedom. The inclusion of this genomic control procedure is not likely to account for the difference in the two approaches because the basic problem with the bootstrap approach concerns using a standard normal distribution centered about 0 to model a non-negative random variable.

We explored the possibility of using a permutation rather than a bootstrap approach to determine whether the addition of another SNP leads to significant improvement in AUC within the Lu-Elston approach. Here, the case-control labels for the additional SNP (one of the 1445) are permuted and an associated *A*_2 _- *A*_1 _difference is computed. One thousand permutations produce an *A*_2 _- *A*_1 _permutation distribution which is compared to the observed *A*_2 _- *A*_1 _in the original data set. If the original *A*_2 _- *A*_1 _exceeds, say, 95% of the empirical *A*_2 _- *A*_1 _distribution, this may be taken as evidence of significant AUC improvement. The approach appears promising but was complicated by the indication of population stratification-the empirical *p*-value distribution was similar to that of the likelihood-ratio test before the stratification adjustment. While the Devlin-Roeder approach to account for stratification may work for a likelihood-ratio test, it is unclear how to proceed for a permutation test. Further, the permutation of labels for just the additional SNP will remove LD with nearby SNPs, which could affect performance.

### Incorporating covariates

Logistic regression easily includes covariate information as additional regressors-the covariates may be discrete or continuous. The Lu-Elston approach toward incorporation of covariates is to first categorize the covariate as a factor (even though it may be continuous in nature). Next, the same multiplicative approach is used to determine the probabilities of observing each combination of covariates and genotypes for cases and controls. From these probabilities the likelihood ratios and ROC curves are constructed as before. In the event the covariates are continuous in nature, such a data transformation entails a loss of information and efficiency.

## Conclusion

The Lu-Elston approach is valuable for developing classification models in the absence of individual-level data. We have applied Lu and Elston's approach for constructing ROC curves and compared it to conventional logistic regression methods. When the assumption of multiplicative effects without interactions among markers is in force there should be little difference between the Lu-Elston and conventional logistic method. The advantages of this conventional approach are the ability to use standard approaches toward model selection based upon log-likelihood differences and a simple way to incorporate covariates via regression.

## List of abbreviations used

AUC: Area under curve; FDR: False-positive rate; GAW16: Genetic Analysis Workshop 16; LD: Linkage disequilibrium; RA: Rheumatoid arthritis; ROC: Receiver operating characteristic; SNP: Single-nucleotide polymorphism; TRP: True-positive rate.

## Competing interests

The authors declare that they have no competing interests.

## Authors' contributions

NJ and GZ together conceived, designed, and review the manuscript. NJ drafted the manuscript and performed the analyses. Both authors read and approved the final manuscript.
